# Widening or narrowing income inequalities in myocardial infarction? Time trends in life years free of myocardial infarction and after incidence

**DOI:** 10.1186/s12963-021-00280-1

**Published:** 2021-12-24

**Authors:** Juliane Tetzlaff, Fabian Tetzlaff, Siegfried Geyer, Stefanie Sperlich, Jelena Epping

**Affiliations:** 1 Medical Sociology Unit, Hannover Medical School, Hanover, Germany; 2 Institute for General Practice, Hannover Medical School, Hanover, Germany

**Keywords:** Myocardial infarction, Incidence, Mortality, Income inequalities, Health expectancies, Germany

## Abstract

**Background:**

Despite substantial improvements in prevention and therapy, myocardial infarction (MI) remains a frequent health event, causing high mortality and serious health impairments. Previous research lacks evidence on how social inequalities in incidence and mortality risks developed over time, and on how these developments affect the lifespan free of MI and after MI in different social subgroups. This study investigates income inequalities in MI-free life years and life years after MI and whether these inequalities widened or narrowed over time.

**Methods:**

The analyses are based on claims data of a large German health insurance provider insuring approximately 2.8 million individuals in the federal state Lower Saxony. Trends in income inequalities in incidence and mortality were assessed for all subjects aged 60 years and older by comparing the time periods 2006–2008 and 2015–2017 using multistate survival models. Trends in the number of life years free of MI and after MI were calculated separately for income groups by applying multistate life table analyses.

**Results:**

MI incidence and mortality risks decreased over time, but declines were strongest among men and women in the higher-income group. While life years free of MI increased in men and women with higher incomes, no MI-free life years were gained in the low-income group. Among men, life years after MI increased irrespective of income group.

**Conclusions:**

Income inequalities in the lifespan spent free of MI and after MI widened over time. In particular, men with low incomes are disadvantaged, as life years spent after MI increased, but no life years free of MI were gained.

**Supplementary Information:**

The online version contains supplementary material available at 10.1186/s12963-021-00280-1.

## Background

Myocardial infarction (MI) is one of the leading diseases in the elderly causing high mortality and serious health impairments [1, 2]. As a result of progressive arteriosclerosis, MI often results in rapid deterioration of cardiovascular health and is associated with long-term impairments in functional health, poor quality of life, and increased risks of recurrent MI and stroke [3–11]. Despite substantial improvements in prevention and treatment, population ageing and increased long-term survival after infarction are expected to lead to an increasing number of individuals living with impaired health after MI [7, 12]. Previous studies mainly focus on the development of incidence risks over time, but research on trends in average lifespan spent without and after MI is sparse and little is known about these trends with regard to social inequalities.

Due to the considerable progress in prevention and therapy, MI incidence has declined in recent decades [13–17]. According to Fries’ morbidity compression hypothesis [18, 19], improved prevention may lead to postponements of disease onset over time and to prolonged periods spent in good health. A recent study reported decreasing incidence rates and increasing age at MI onset among men, pointing towards morbidity compression [20]. On the other hand, improvements in therapy have increased survival rates after MI substantially [10, 13, 21, 22], thus leading to prolonged periods spent after MI and contributing to an expansion of morbidity [23]. For Germany, both decreasing incidence rates and mortality rates after MI onset have been reported [16, 24]. Therefore, it largely remains unclear whether a compression or an expansion has taken place among the German population and whether these developments depend on socioeconomic characteristics.

Numerous studies have emphasised the important role of social status in determining differences in the risk of MI between population subgroups [25–30]. Social inequalities in MI incidence and MI mortality were also reported for Germany [31–33]. The development of social inequalities in MI incidence over time in Germany has rarely been examined. One of the few existing studies found narrowing income inequalities in MI incidence risks in men aged 18 years and older, while no changes were reported in women [34].

However, research is still lacking on how the average lifespan spent free of MI and life years affected by MI developed over time and whether these trends differ between social subgroups. This study steps into this gap by investigating time trends in the number of life years free of MI and after MI incidence by income group. Special attention is paid to whether these time trends differ between income groups, causing narrowing or widening inequalities over time. The analyses are based on the data of a large German health insurance provider. The high case numbers make it possible to analyse the interplay between MI incidence and mortality developments over time in different income groups. In detail, the study was guided by the following research questions:

- Are there income inequalities in MI incidence risks and mortality risks after MI and without MI? How did these inequalities develop over time?
- Are there income inequalities in the number of life years spent free of MI and after MI incidence? Have these inequalities widened or narrowed over time?

## Methods

### Data

The study is based on claims data of a large German statutory health insurance provider, the AOK Niedersachsen (AOKN) insuring approximately one third of the total population of the federal state Lower Saxony [35]. The data were collected for accounting purposes and include in- and outpatient diagnoses coded according to the International Classification of Disease 10th revision (ICD-10), demographic and socioeconomic information and the date of death for all individuals deceased during the observation period. Previous analyses have shown that the data are representative for the total German population in terms of sex and age distribution, while individuals with higher incomes are underrepresented [36].

In Germany, health insurance (private or publicly-funded statutory insurance) is mandatory for all residents. Below a legally determined income threshold, private health insurance is only accessible to self-employed individuals and to government officials. Since this income limit is considerably higher than the average income in Germany, the vast majority (approximately 90%) is insured with one of the providers of the statutory health insurance system [37]. Within the statutory health insurance system, the health care coverage is the same for all individuals. Since all rendered medical services have to be reported to the insurer as a prerequisite for payment, claims data of the statutory health insurances depict health care activities fairly complete.

The data were available for the years 2005 to 2017. As the number of incident cases in MI and deaths after MI by income group is limited, single years of observation were combined into time periods. Therefore, time trends in incidence and mortality were analysed by comparing the two time periods 2006–2008 and 2015–2017. Since the number of deaths after MI and MI incident cases are limited in younger ages, the analyses are based on the insurance population aged 60 years and older.

### Incidence

MI incidence was determined on the basis of the ICD-10 inpatient diagnoses I21.0 to I21.9. Incident cases were defined for individuals having a MI diagnosis in 2006–2008 or 2015–2017. To distinguish between incident and recurrent cases, look-back periods of 1 year preceding this diagnosis were applied that had to be free of MI in incident cases. Recurrent cases were excluded from all analyses performed in this study.

### Income

In Germany, employers are obligated to report the pre-tax incomes of their employees to the statutory health insurance provider. Furthermore, the data contain information on pension payments received from the German Pension Fund and on unemployment benefits. Income groups were defined relative to the average pre-tax income in Germany of a given year reported by the Federal Statistical Office. In this way the grouping is taking varying income levels over time into account. Since the number of incident cases and deaths within the different subgroups is limited, the study population was classified into two groups. The low income group includes all individuals with an annual income ≤ 60% of the average income in Germany. Individuals having > 60% of the average income were assigned to the higher income group. Furthermore, incomes were adjusted for inflation, which allows direct comparability between years, as purchasing power is kept constant over time. Taking both periods together, the proportion of individuals with missing information on income amounted to 8%.

### Statistical analyses

The analyses are based on multistate survival analyses using a three-state illness-death model. Within this model, three types of events are possible, which are defined by the following transitions: (1) MI-free to MI (the event of MI incidence), (2) MI-free to death (death without MI), and (3) MI to death (death after MI incidence).

In a first step, general *income inequalities in incidence and mortality risks* were analysed by proportional hazard models with constant baseline hazard over time. The models were stratified by sex and controlled for time period and age. *Time trends of income inequalities in incidence and mortality risks* were analysed in two steps: First, trends within income group were estimated separately for income group and sex. The models include a covariate for time period. Furthermore, changes in income inequalities in incidence and mortality risks were analysed. These analyses were based on interaction models (time period*income group) and were stratified by sex. Finally, *time trends in the number of life years spent free of MI and after MI* were calculated by multistate life table (MSLT) analyses. The development of the expected number of life years spent free of MI and after MI depends on the complex interplay between the development of incidence, mortality after MI and mortality without previous MI. Therefore, age-specific hazard rates of all three transitions have to be included in the MSLT analyses. These age-specific hazard rates were predicted from proportional hazard models with constant baseline hazard over time and included as input for the MSLT. The models are stratified by sex, income group, and time period and were fitted separately for each type of transition. All models contain a covariate for age in single-years age groups (centered to the middle of the age interval 60 to 95+ and included as second-degree polynomial), which varies with calendar year. The data management and the estimation of the survival models were performed with Stata MP 14.2 [38], the MSLT were calculated with R 3.5.1 [39]. All confidence intervals were estimated by drawing 1000 bootstrap samples (with replacement).

## Results

In both periods, 35,037 MI incidences with 4,120,195 person-years at exposure and 169,980 deaths without MI with 4,120,227 person-years at exposure were observed. Among the MI incident individuals, a total number of 12,771 deaths occurred over an exposure time of 36,696 person-years. Age-unadjusted crude incidence rates per 1000 person-years of exposure remained largely stable between periods in men (10.99 in 2006–2008 to 11.03 in 2015–2017) but decreased in women (6.95 to 6.64). The crude death rates after MI decreased for both sexes (335.10 to 297.32 in men and 409.01 to 350.00 in women) (Table 1). Since the study population is limited to individuals aged 60 years and older and includes many individuals after retirement age, a considerable proportion of the individuals belong to the low-income group. This applies particularly to women reflecting the overall lower income level than in men (Table 1).

**Table 1:** Descriptive statistics on the number of insured individuals, exposures in person-years, and events by type of transition, sex, income group, and period

	Men			Women		
	Low	Higher	Total	Low	Higher	Total
**Period 1: 2006–2008**						
**MI incidence**						
*N*	159,464	126,681	286,145	342,983	101,232	444,215
Exposures	431,720	342,316	774,036	948,054	274,743	1,222,797
Events	5069	3445	8514	6800	1703	8503
Rate^1)^	11.74	10.06	10.99	7.17	6.20	6.95
**Death without MI**						
*N*	159,464	126,681	286,145	342,983	101,232	444,215
Exposures	431,722	342,320	774,041	948,057	274,743	1,222,800
Events	20,276	12,202	32,478	39,125	8829	47,954
Rate^1)^	46.97	35.65	41.96	41.27	32.14	39.22
**Death after MI**						
*N*	5150	3500	8650	6828	1722	8550
Exposures	5308	3619	8927	6525	1647	8172
Events	1844	1148	2992	2821	676	3497
Rate^1)^	347.40	317.21	335.16	432.33	410.44	409.01
**Period 2: 2015–2017**						
**MI incidence**						
*N*	199,895	132,857	332,752	339,396	111,501	450,897
Exposures	537,024	355,987	893,011	929,177	301,173	1,230,350
Events	6514	3334	9848	6422	1750	8172
Rate^1)^	12.13	9.36	11.03	6.91	5.81	6.64
**Death without MI**						
*N*	199,895	132,857	332,752	339,396	111,501	450,897
Exposures	537,030	355,998	893,028	929,183	301,176	1,230,358
Events	27,432	11,680	39,112	40,017	10,419	50,436
Rate^1)^	51.08	32.81	43.80	43.07	34.60	41.00
**Death after MI**						
*N*	6635	3431	10,066	6468	1769	8237
Exposures	7328	3629	10,958	6803	1836	8640
Events	2257	1001	3258	2390	634	3024
Rate^1)^	308.00	275.83	297.32	351.32	345.32	350.00

^1^Crude rate per 1000 person-years of exposure

### Income inequalities in incidence and mortality risks

Overall, income inequalities in MI incidence and mortality risks are more pronounced in men than in women (Table 2). Compared to the low-income group, men in the higher-income group had a 14% lower risk of MI incidence, a 26% lower mortality risk without previous MI, and an 18% lower risk of death after MI. Among women, inequalities between income groups were small and were most pronounced in mortality without MI (HR = 0.94) (Table 2).

**Table 2:** Risks (HR) of myocardial infarction incidence, death without myocardial infarction, and death after myocardial infarction of the higher-income group compared to the low-income group by sex

	MI incidence	Death without MI	Death after MI
**Men**			
Low	1	1	1
Higher	0.86 (0.84–0.89)	0.74 (0.73–0.75)	0.82 (0.78–0.87)
**Women**			
Low	1	1	1
Higher	0.99 (0.96–1.03)	0.94 (0.93–0.96)	0.98 (0.92–1.04)

HR Hazard Ratio; 95%-CI bootstrapped (with replacement) using 1000 replications; all analyses are controlled for age in single-year age groups (as second-degree polynomial) and for time period

### Time trends in incidence and mortality risks

**Fig. 1:**
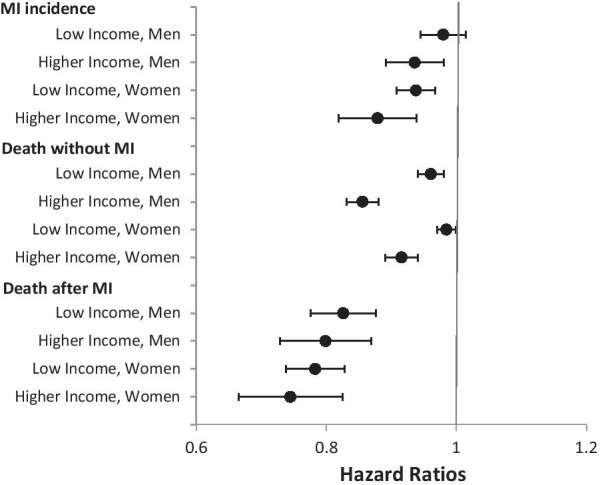
Time trends in risks of myocardial infarction incidence, death without myocardial infarction, and death after myocardial infarction by sex and income group (Hazard Ratios of time periods, reference: period 1 (2006–2008)). Hazard Ratios estimated by multistate survival models; 95%-CI bootstrapped (with replacement) using 1000 replications; all analyses are controlled for age in single-year age groups (as second-degree polynomial); MI myocardial infarction

Incidence risks decreased in all groups, with the exception of low-income men, for whom the risks remained quite constant over time (Fig. 1). Mortality risks without MI decreased in both income groups and for both sexes. The same holds for death after MI, where the decreases over time were strongest. In general, the decline of event risks tends to be stronger in the higher- than in the low-income group (Fig. 1).

**Fig. 2:**
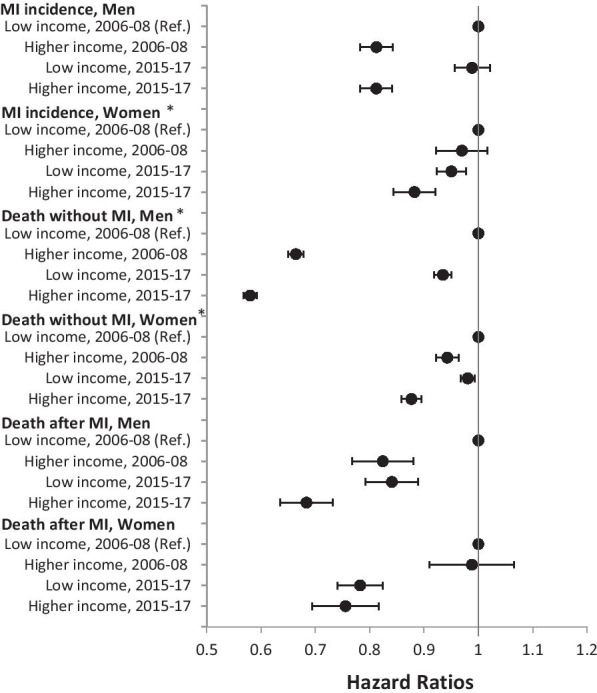
Income inequalities in risks of myocardial infarction incidence, death without myocardial infarction, and death after myocardial infarction by sex (Reference: low income, period 1 (2006–2008)). Hazard Ratios were estimated from multistate survival models including the interaction income group*period; 95%-CI bootstrapped (with replacement) using 1000 replications; * significant interaction term (income group*period) based on 95% CIs; all analyses are controlled for age in single-year age groups (as second-degree polynomial); MI myocardial infarction

With respect to income inequalities in MI incidence risks, the gap tended to widen in women but remained largely constant in men. Widening income inequalities were also found in mortality without MI in both sexes. Despite a tendency towards increasing inequalities in death after MI, the change over time remained limited leading to rather constant inequalities over time (Fig. 2).

### Time trends in life years free of MI and after MI

Calculations of the number of life years free of MI and after MI incidence are based on age-specific hazard rates predicted by multistate survival models of the three transitions to smooth fluctuations. Additional files 1 and 2 allow for a comparison between the observed rates and the predicted rates. Overall, the predicted rates fit the observed rates pretty well. Due to the limited number of cases at older ages, the observed rates fluctuated more strongly above age 80. These fluctuations were smoothed by the predicted rates (Additional files 1 and 2).

**Fig. 3:**
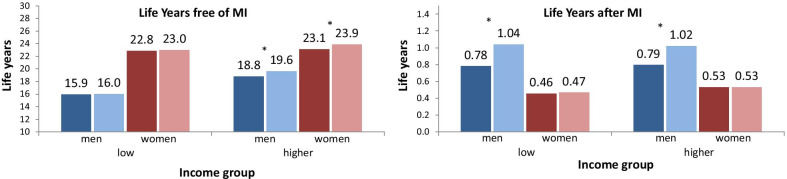
Expected number of life years free of myocardial infarction and after myocardial infarction at age 60 by sex, income group and time period. Period 2006–2008 in darker colours, period 2015–2017 in lighter colours, 95%-CI bootstrapped (with replacement) using 1000 replications; * Significant difference in the number of life years between the two periods based on 95%-CIs (bootstrapped, using 1000 replications); MI myocardial infarction

Figure 3 shows the expected number of remaining life years free of MI and after MI at age 60. While the expected number of MI-free life years in the first period is similar in women of both income groups, considerable inequalities were found among men. Between the periods, the number of MI-free life years increased by 0.8 years for men of the higher-income group (18.8 to 19.6), while it remained stable in low-income men. Among women, these time trends are very similar, since only women with higher incomes benefitted from an increase in life years free of MI (23.1 to 23.9). The number of life years after MI incidence is almost identical in both income groups. This applies to both sexes. Due to the increased survival after MI, the number of life years after MI increased in men of both income groups (0.78 to 1.04 in the low- and 0.79 to 1.02 in the higher-income group). For women, no changes over time were found.

## Discussion

This study investigated time trends in income inequalities in MI-related health expectancies of individuals aged 60 years and older based on German health insurance data. The study has shown that income inequalities in the expected length of life spent free of MI and after disease onset have widened over time. Driven by the stronger decline in incidence in women and mortality risks without previous MI in both sexes, the number of MI-free life years increased among the higher-income group while no life years free of MI were gained among individuals belonging to the low-income group. Due to the clear decline in mortality rates after MI, the expected lifespan after incidence increased for men irrespective of income group. As no MI-free life years were gained among men with low incomes, the relative proportion of life years after MI increased more strongly than in men with higher incomes. In contrast, the number of life years after MI remained constant in women as the decreases in incidence rates were counterbalanced by the declining mortality rates after MI.

Our results are in line with the general trend towards declining MI incidence rates and improved survival after MI reported in many other studies [10, 13–17, 21, 22]. While the trend towards decreasing incidence and general mortality leads to gains in life years spent free of MI, the decline in mortality after MI fosters extended periods in ill health. Studies aimed at analysing whether compression or expansion has occurred over time need therefore to focus on the interplay of trends in incidence and mortality without and after disease onset. With respect to income inequalities, our findings indicate that differences in MI incidence and mortality between income groups are substantial in men but less pronounced in women, which is in accordance with a recent study on inequalities among individuals aged 18 years and older of the same insurance population [34]. However, the analyses also show that time trends in MI incidence and mortality risks tend to differ by income group, leading to different trends in life years spent free of MI and after MI incidence between income groups. Due to the decline in mortality risks after MI among men, the number of life years spent after MI increased in both income groups. This expansion was most pronounced among men belonging to the low- income group as no life years free of MI were gained. Among women, the number of life years after MI remained largely the same in both income groups. However, due to the increase in MI-free life years, the relative proportion of years spent after MI decreased among women belonging to the higher-income group, pointing towards morbidity compression. Due to these differences between income groups, inequalities also widened in women.

The trend towards widening inequalities may be driven by differing trends in cardiovascular risk factors and health-related behaviour. For Germany, decreasing prevalence of hypertension, hyperglycaemia, hyperlipidaemia, smoking, and physical inactivity have been reported [40–42]. So far, research investigating changes in cardiovascular risk factors with respect to socio-economic status (SES) group among the German population is rare, but increasing inequalities were reported for smoking in men [43] and physical inactivity in both sexes [44], which may have fostered the stronger increase in MI-free life years among higher-income groups. Sharing similar risk factors and pathomechanisms, increasing inequalities in stroke-free life years have also been found for German men [45]. Over the last decades, the income gap in Germany has been rising constantly [46]. This suggests that in the future a growing proportion of the population will fall into the low-income group and will therefore be particularly disadvantaged in terms of health and life expectancy [47]. This additionally exacerbates the effect of growing inequalities in health expectancies and stresses the importance of further research on trends in risk factors and public health efforts aiming to reduce health inequalities and harmful health behaviour in vulnerable social groups.

Our findings on income gradients are in line with a previous study of Geyer et al. based on the same data source that reported strong inequalities in MI incidence among men but not in women [34]. With respect to differences in time trends in the low and higher income groups, however, the results differ for men as the strongest declines in incidence risks were found in men with low incomes [34]. This discrepancy may be explained by differences in the study population. While Geyer et al. focused on the full age range of the adult population (age 18 and above), the present study examines population health among the elderly and therefore depicts trends in the population subgroup carrying the main burden of MI morbidity. The results of the two studies suggest, however, that premature MIs also contribute significantly to inequalities in cardiovascular health in the population and that time trends between premature MIs and MIs in the main disease age may differ. However, due to the limited number of incident cases below age 60, differences in trends in the number of life years free of MI and after incidence could not be analysed in this study. Further research should investigate these differences in more detail.

Previous research has shown that trends in incidence and the general mortality after MI differ between ST segment elevation (STEMI) and non-STEMI MIs [13]. However, since the ICD-10 classification system does not allow a precise distinction between STEMI and non-STEMI MIs, these differences could not be considered in our study. Previous results also suggest that the degree of long-term impairments caused by MI may not be the same between SES groups as functional recovery was found to be greatest in patients with high SES [48]. Including analyses on social inequalities in functional impairments or quality of life after MI would have provided a deeper insight into trends in the burden of MI. However, as claims data do not contain the information needed to determine one of the common indices for measuring long-term functional impairments or self-perceived health-related quality of life, this issue could not be addressed.

### Strengths and limitations

The study is based on large numbers of incident cases and deaths with and without previous MI, which allowed robust estimates of the risks of all three events, even if the analyses are performed separately for income group and sex. The precise information on dates of MI and death available in the data allows to determine the exact order of events, which is necessary when performing multistate survival analyses. Moreover, the data include the complete insurance population, irrespective of health condition. Therefore, the analyses are unaffected by health-related non-response, which may occur in survey data if individuals do not participate in the study due to ill health.

With respect to age and sex distributions, the data are representative for the total German population [36] but differ in terms of social structure as individuals with high incomes are underrepresented. This has been taken into account since all analyses are stratified by income group. As premium payments depend on income level, information on individual income is available for the insured individuals being an active part of the labour force, receiving unemployment benefits, or receiving pension payments from the German Pension Fund. Individuals who are not part of the labour force and are not retired were excluded from the analyses as information on income is missing. However, as the study population is limited to the age group 60 and above and the majority belongs to the group of retired individuals, income information is available for the vast majority of the study population (92%).

In our study, recurrent cases of MI were identified using a look-back period of one year. Previous research shows that most of the recurrent MIs occur in the first year after the first MI [10, 49] and it can therefore be assumed that a substantial share of recurrent MIs was identified. Nevertheless, using this approach a certain proportion of recurrent MIs cannot be identified. Longer look-back periods would have reduced this proportion. However, a recent study has shown that there is always a trade-off between the length of look-back periods and the resulting shift in the socio-demographic structure of the insurance population [50]. This shift is caused by more rigorous preconditions on the length of the observation time, which must be met when longer look-back periods are applied. Therefore, individuals with shorter insurance histories would have to be excluded, leading to an increasingly selective study population. However, the shift in the sociodemographic distribution remains minor when look-back periods of one year are applied [50].

## Conclusions

This study shows that the general trend of declining MI incidence and mortality rates is accompanied by increasing social inequalities in the length of life free of MI and in the life years affected by MI. While incidence and mortality risks declined in all income groups, decreases were most pronounced in the higher-income group. This led to a growing disadvantage of individuals with low SES. This applies especially to men with low incomes, as the lifespan spent after MI expanded but no healthy years were gained over time. Further research is needed on social inequalities in long-term functional limitations and quality of life after MI and on whether the burden of these impairments developed similarly in the different SES groups.

## Supplementary Information

**Additional file 1**. Observed and predicted hazard rates of (a) incidence of myocardial infarction, (b) death without myocardial infarction, and (c) death after myocardial infarction for men of the low-income group (≤60% of the average income in Germany, left) and of the higher-income group (>60% of the average income in Germany ,right) by period. Predicted hazard rates are derived from proportional hazard multistate survival models with constant baseline hazards; all survival models are controlled for age in single-year age groups (as second-degree polynomial)

**Additional file 2.** Observed and predicted hazard rates of (a) incidence of myocardial infarction, (b) death without myocardial infarction, and (c) death after myocardial infarction for women of the low-income group (≤60% of the average income in Germany, left) and of the higher-income group (>60% of the average income in Germany ,right) by period. Predicted hazard rates are derived from proportional hazard multistate survival models with constant baseline hazards; all survival models are controlled for age in single-year age groups (as second-degree polynomial)

## Data Availability

The datasets generated and analysed during the current study are not publicly available due to protection of data privacy of the insured individuals by the Statutory Local Health Insurance of Lower Saxony (AOK Niedersachsen).

## Abbreviations

MI: Myocardial infarction
AOKN: AOK Niedersachsen
ICD-10: International Classification of Disease 10th revision
MSLT: Multistate life table
STEMI: ST segment elevation
SES: Socio-economic status
