# Supplementary material for: Widening or narrowing income inequalities in myocardial infarction? Time trends in life years free of myocardial infarction and after incidence
Source: Popul Health Metr. 2021 Dec 24;19:47. doi: 10.1186/s12963-021-00280-1 (PMC8709953; doi:10.1186/s12963-021-00280-1)
Supplement: Supplementary file 1 — Additional file 1. Observed and predicted hazard rates of (a) incidence of myocardial infarction, (b) death without myocardial infarction, and (c) death after myocardial infarction for men of the low-income group (≤60% of the average income in Germany, left) and of the higher-income group (>60% of the average income in Germany ,right) by period. Predicted hazard rates are derived from proportional hazard multistate survival models with constant baseline hazards; all survival models are controlled for age in single-year age groups (as second-degree polynomial) [file 12963_2021_280_MOESM1_ESM.pdf]

Additional file 1 Observed and predicted hazard rates of (a) incidence of myocardial infarction, (b) death without myocardial infarction, and (c) death after myocardial infarction for men of the low-income group (≤60% of the average income in Germany, left) and of the higher-income group (>60% of the average income in Germany ,right) by period

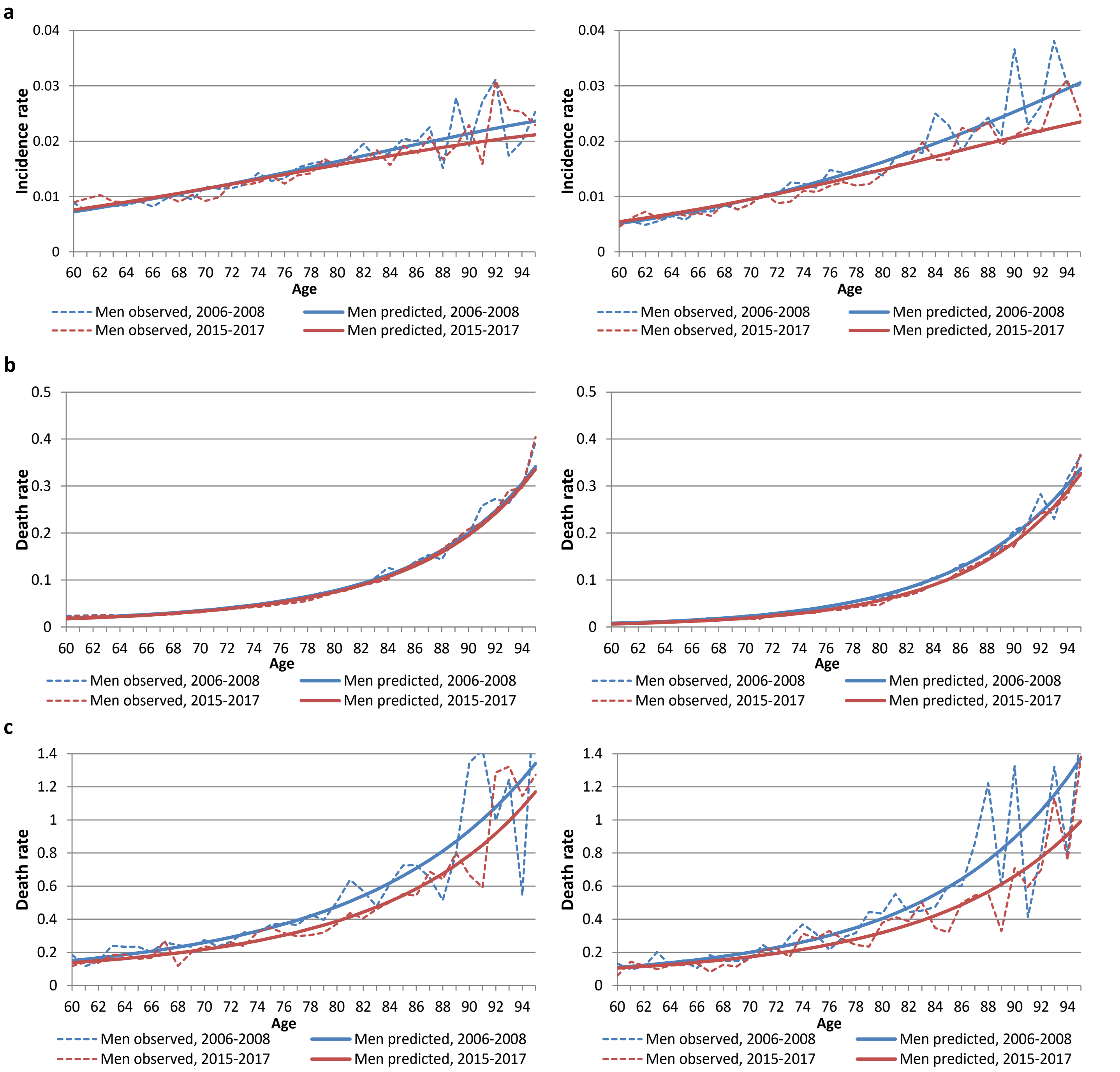

Predicted hazard rates are derived from proportional hazard multistate survival models with constant baseline hazards; all survival models are controlled for age in single-year age groups (as second-degree polynomial)
